# Expanding the perspective of translational medicine: the value of observational data

**DOI:** 10.1186/1479-5876-10-61

**Published:** 2012-03-27

**Authors:** Michael N Liebman, Francesco M Marincola

**Affiliations:** 1 Strategic Medicine, Inc, 231 Deepdale Drive, Kennett Square, PA 19348, USA; 2 National Institutes of Health, Bethesda, MD, USA

## Abstract

In 2003, the Journal of Translational Medicine was launched to foster the publication of high quality research in both "bench-to-bedside" as well as ex vivo human observation. In spite of the success of several large-scale observational studies, e.g. Framingham Heart Study, the opportunity to expand upon the ex vivo human observation has remained limited within the field of translational medicine. We believe that this presents a significant opportunity that merits consideration in both the planning and analysis of large scale observational studies and can contribute greatly to expanding our approaches in translational medicine

## Editorial

The Journal of Translational Medicine (JTM) was launched in 2003 with an editorial titled "Translational Medicine: A two-way road". In the following years, through the dedicated efforts of its editorial and advisory boards, dedicated reviewers and excellent researcher submissions, it has established a solid reputation in the area of translational medicine although, by necessity (based on submission), it has primarily represented one-way on the two-way road, namely that of moving from Bench-to-Bedside. As outlined in that original editorial, this is actually not surprising, but it is suggestive that there is opportunity to examine how to "develop traffic heading in the other direction", from Bedside to Bench. In this editorial we focus on the use of large epidemiological studies and the specific opportunity for them to play a significant role in translational medicine.

In July, 2003, the editorial stated that "Translational Research would be most useful to the scientific community at large if journals would foster specific interest for the publication of ex vivo human observation". We believe that an important approach towards this goal might be through the detailed analysis and interpretation of large scale observational data sets, collected with the same quality standards as applied in clinical studies or clinical trials, and used to identify new questions and lead to potential hypotheses that foster further experiment and analysis.

Observational studies differ from standard clinical studies in that they may involve the collection of observations of individuals over an extended period of time without focusing on the specific comparison of "treated" versus "untreated" groups [1]. Such studies may involve the study of behaviors, responses, etc. in populations where randomized experimental design might violate ethical standards, e.g. risk of smoking on pregnancies, or may be impractical, e.g. the relationship between a specific treatment and potential side-effects that may be rare [2]. They focus on establishing association, not proving cause and effect, but can be invaluable for use in the design of more conventional, controlled clinical studies. We would suggest, however, that for observational studies to be considered within JTM's definition of clinical practice, there are several qualities that such a study needs to address in its design and implementation and we outline these below. There are well known examples of such studies: the Framingham Heart Study [3], The Nurses Health Study [4], the Women's Health Initiative (which includes both conventional clinical studies as well as an observational study) [5] and the Surveillance Epidemiology and End Results Database (SEER) [6]. While these examples are primarily of US origin, international large scale observational studies need to also be acknowledged as to their relevance in Translational Medicine, notably the Busselton Health Study (Australia) [7] and the China-Cornell-Oxford Study [8].

In this manner, the identification of key "bedside" issues that exist within such large population-based studies can form the basis for many more directed studies which can lead to results of immediate clinical utility. While conventional analytical methods, including parametric and non-parametric statistical analysis, can identify significant trends and research questions, more recent approaches, including social network analysis, can be used to identify hypotheses and patterns within these complex data sets that may currently elude more conventional investigations. This follows the adage of "let the data speak for itself" without always having a pre-determined way to examine and interpret it, and would be particularly useful in integrating and analyzing multi-disciplinary data resources. In addition, this approach can contribute to helping clinicians and clinical researchers realize the true complexity that underlies the observational data and support further communication of "bedside"-focused needs with the "bench-based" research community.

It has been pointed out that observational studies are sometimes confused as providing an adequate basis for "evidence-based" medicine and it is clear that such studies, as listed above, have yielded risk algorithms and potential contraindications that have been used in clinical practice, possibly without adequate validation [9]. We do not promote such use but actually support the concept the whether research begins in the laboratory or in an observational study, the potential conclusions that can impact clinical practice, i.e. translational research, still need, in both cases, full clinical validation through the randomized clinical trial process. We readily accept this as the next stage in moving "bench to bedside" but do not always utilize this approach in moving from "observational study/bedside to bench to bedside". Thus the Framingham Cardiac Risk Algorithms and the Gail Model for Breast Cancer Risk (and its derivatives) all require the same rigor in validation that analogous results from analyzing a biomarker in a pathway in an animal model also deserve before moving into the clinic.

We do believe that certain guidelines should be established to support the development and operation of observational studies towards their role in translational medicine:

1. As most observational studies are longitudinal in nature, a data collection instrument, i.e. questionnaire, must be developed and maintained for consistency through the study

2. Addition of new data elements and/or definitions should be incorporated in follow on versions of the study to support meta-analysis across the study while allowing for new knowledge to be incorporated (see [3] and [4])

3. Development of criteria to deal with the potential biases in patient-generated vs physician/nurse generated data needs to be validated

4. Development of processes for data validation to be applied across the study period

5. Potentially provide access to data acquisition instrument and definitions to support meta-data analysis across studies while maintaining potential proprietary concerns about the instrument

6. Development of statistically significant populations to support application of parametric and non-parametric data analysis (see [3-9])

7. Extend analysis of data beyond simple statistical profiling to support the development of hypotheses leading to more conventional clinical studies

It may be an interesting example for the research community in translational medicine to expand the challenge to broaden the community to include epidemiologists and systems modelers who focus on identifying and extracting patterns from retrospective, ex vivo observations. All this is intended to help our ultimate goal of improving healthcare for the patient and the physician.

## Competing interests

The authors declare that they have no competing interests.
